# Cognitive and memory training in adults at risk of dementia: A Systematic Review

**DOI:** 10.1186/1471-2318-11-55

**Published:** 2011-09-25

**Authors:** Nicola J Gates, Perminder S Sachdev, Maria A Fiatarone Singh, Michael Valenzuela

**Affiliations:** 1 School of Psychiatry, University of New South Wales, RandwickNSW 2031, Australia; 2 Brain and Aging Research Program, University of New South Wales, Randwick NSW 2031, Australia; 3 Regenerative Neuroscience Group, School of Psychiatry, University of New South Wales, Randwick NSW 2031, Australia; 4 Neuropsychiatric Institute, Prince of Wales Hospital, Randwick NSW 2031, Australia; 5 Exercise Health and Performance Faculty Research Group, Sydney Medical School, The University of Sydney, Lidcombe NSW 2141, Australia; 6 Hebrew Senior Life, Boston, MA, and Jean Mayer USDA Human Nutrition Research Center on Aging at Tufts University, Boston 02130, MA, USA

## Abstract

**Background:**

Effective non-pharmacological cognitive interventions to prevent Alzheimer's dementia or slow its progression are an urgent international priority. The aim of this review was to evaluate cognitive training trials in individuals with mild cognitive impairment (MCI), and evaluate the efficacy of training in memory strategies or cognitive exercises to determine if cognitive training could benefit individuals at risk of developing dementia.

**Methods:**

A systematic review of eligible trials was undertaken, followed by effect size analysis. Cognitive training was differentiated from other cognitive interventions not meeting generally accepted definitions, and included both cognitive exercises and memory strategies.

**Results:**

Ten studies enrolling a total of 305 subjects met criteria for cognitive training in MCI. Only five of the studies were randomized controlled trials. Meta-analysis was not considered appropriate due to the heterogeneity of interventions. Moderate effects on memory outcomes were identified in seven trials. Cognitive exercises (relative effect sizes ranged from .10 to 1.21) may lead to greater benefits than memory strategies (.88 to -1.18) on memory.

**Conclusions:**

Previous conclusions of a lack of efficacy for cognitive training in MCI may have been influenced by not clearly defining the intervention. Our systematic review found that cognitive exercises can produce moderate-to-large beneficial effects on memory-related outcomes. However, the number of high quality RCTs remains low, and so further trials must be a priority. Several suggestions for the better design of cognitive training trials are provided.

## Background

Development of preventative strategies for Alzheimer's dementia (AD) is an international priority, with prevalence rates projected to increase by over 75% in the next quarter of a century [1]. One approach to reduce the prevalence of AD is to develop strategies to delay its onset in healthy individuals or those at risk of developing dementia. Prospective cohort studies have found that participation in mentally-stimulating activities is associated with a lower incidence of AD [2] and even late-life mental activity exhibits a dose-dependent inverse relationship with dementia risk, independent of early life experiences [3]. Consequently, it is possible that participation in complex mental activities at older age may offer protection from cognitive decline and hence mitigate dementia risk.

Cognitive training provides structured practice of complex mental activity in order to enhance cognitive function [4], and has attracted intense public, commercial and scientific interest. Unfortunately, cognitive training interventions have been frequently mislabelled or conflated with other therapies, despite important theoretical distinctions between compensatory cognitive rehabilitation, general cognitive stimulation and cognitive training [5-7]. For example, the non-specific umbrella terms 'cognitive intervention' [8], 'cognitive enrichment' [9] and 'cognitive rehabilitation' have been applied to multidomain cognitive training [10-12] as well as training in memory strategies [13]. 'Cognitive stimulation' has been used to refer to interventions ranging from generic topical discussions [14], executive exercises and memory strategy training [15]. Given the confusion of terms, an operational definition has been advanced which delineates cognitive training from other interventions [16]: 1) repeated practice, 2) on problem activities, 3) using standardized tasks, and 4) that target specified cognitive domains.

Cognitive training can be further distinguished to include training in applied memory strategies versus repetitive cognitive exercises [7]. Training in memory strategies involves the instruction and practice of techniques to minimize memory impairment and enhance performance, and involves learning and practicing strategies such as the method of loci, mnemonics, and visual imagery [17,18]. In contrast, cognitive exercise requires the repeated practice of targeted cognitive abilities in a repetitions-sessions format analogous to 'reps-sets' regimes in physical resistance training: users typically carry out a number of iterations of a cognitive task in one session, then continue to new tasks in the next session, and eventually return to further train the original task at a harder level in future sessions (i.e., staircase design). Recently, several software applications have been developed that implement cognitive exercises on computer [19,20].

Although cognitive exercises and memory strategies are structurally distinct, they have often been analysed together. A Cochrane review of 32 training trials up to the year 2007, concluded that none of the effects could be attributed specifically to cognitive training, however, only memory training data from 24 trials were pooled for analysis, and the analysis did not include results from cognitive exercise trials of problem solving and speed of information processing [4]. Similarly a review of memory strategy training in healthy and mild cognitive impairment (MCI) individuals [18] combined results from two trials of cognitive exercises [11,19] with 22 trials of memory strategy training and found no specific effects of training. Furthermore, mixed results were also obtained in a systematic review of cognitive interventions in MCI which included training in both memory strategies and cognitive exercises [21]. In addition, many of the trials included uncontrolled interventions such as use of external memory aids or relaxation therapy [22]. Prior reviews have therefore not appropriately distinguished between types of cognitive training, potentially obscuring clinically-relevant effects. Furthermore, a lack of differentiation between cognitive exercises and training in memory strategies, and the inclusion of multiple other therapies with cognitive training, may have also contributed to mixed findings.

By contrast, a meta-analysis of longitudinal RCTs of cognitive training (as defined here) in cognitively healthy adults demonstrated efficacy on primary cognitive outcomes [23]. However, whether operationally-defined cognitive training can be as effective at slowing the rate of cognitive decline after clinical signs are apparent is not clear. MCI is a diagnostic term applied to those individuals with high risk of developing dementia and in the intermediate stage between normal cognitive function and dementia [24,25]. MCI increases the risk for dementia, with diagnosed individuals progressing at rates of 12-15% per year compared to 1-2% of the general population [26]. Cognitive training at this preclinical stage may potentially prevent or delay disease onset, reducing this high conversion rate.

The purpose of this systematic review was therefore to identify all relevant clinical trials of defined cognitive training in individuals with MCI in order to: a) determine the overall efficacy of cognitive training in at risk individuals; b) compare outcomes between cognitive exercises and memory strategy training; c) examine the issue of generalisation of training; d) identify and discuss limitations of current research, and e) provide recommendations for future research.

## Methods

### Search Strategy

To identify relevant research trials Medline (1996-18 March 2011), EMBASE (1980-18 March 2011), CINAHL (1980-18 March 2011) and PsychINFO (1984-18 March 2011) databases were searched by NG. The key search term ["cognitive training"] was supplemented with ["cognitive intervention"] ["cognitive rehabilitation"] ["cognitive stimulation"] [cognitive enrichment'] ["memory training"] and ["memory rehabilitation"]. The sample population of interest was the elderly with cognitive impairment but no dementia, and in order to identify this group multiple search terms were entered ["MCI" or "mild cognitive impairment"][ "pre-dementia"] ["mild cognitive disorder"] [" age associated cognitive decline"]or ["cognitive impairment no dementia"]. Combined intervention and population terms were searched in "All Fields", and identified papers were reviewed (title/abstract) by NG to identify potentially relevant trials and this was supplemented by reviewing the reference list of retrieved trials.

### Inclusion criteria

Studies were selected from the initial search if they met the following criteria i) described a cognitive training intervention consistent with our definition ii) were a full length article published in a peer reviewed English language journal iii) study design was a randomised controlled trial (RCT), or non-randomised (NRCT) or uncontrolled clinical trial (UCT) iv) sample population was defined as having MCI or in a mixed sample the data for MCI was available separately [16] v) no training in external memory aids and vi) baseline and post intervention results on at least one cognitive outcome measure.

### Appraisal of Study Quality and Data extraction

Included studies were individually scored on their published adherence to the CONSORT 2001 reporting criteria for clinical trials (http://www.consort-statement.org) accessed 17 March 2011). Key information was extracted by two reviewers (NG and MV) onto a standard template and any differences resolved by consensus with other authors. Additional non published outcome data were received from the authors of two trials [27,28].

### Analytical Approach

Data were extracted for the description of methodology and common outcomes of each trial. A quantitative meta-analysis was our primary goal if a sufficient number of quality studies using homogeneous interventions and outcomes were identified. Our secondary goal was to calculate effect sizes, statistical power along with clinical relevance in all studies and describe important trial characteristics such as: cohort, intervention type, training delivery, volume of training, outcome measures, and follow-up. Relative effect sizes for RCTs and NRCTs were calculated as a difference of change scores with pooled baseline standard deviation (Coe's Calculator retrieved May 5, 2009 from http://www.cemcentre.org/evidence-based-education/effect-size-calculator ). Hedge's bias corrected relative effect sizes were obtained with 95% confidence intervals as this method adjusts for small sample size. Post-hoc power calculations were calculated with GPower Analysis Version 2.0 [29].

## Results

### Search Results

**Figure 1:**
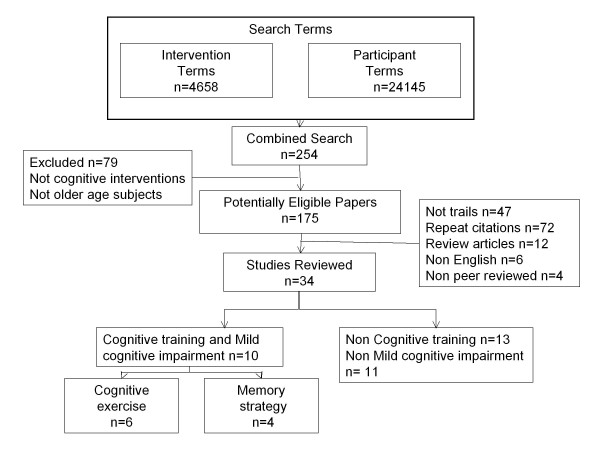
**Flow of eligible trials into review**.

A summary of eligible articles into the review is presented in Figure 1. The combined search of intervention terms AND population terms yielded 175 potentially eligible papers. The abstracts were reviewed, providing a final total of 34 studies, agreed upon by all authors, which were reviewed in full to determine suitability for inclusion. Ten studies met our criteria for cognitive training and MCI: 6 trials of cognitive exercises (3 RCTs, 2 UCTs, 1 NRCT) and 4 training in memory strategies (2 RCTs, 2 NRCTs). A number of studies were excluded because the sample was mixed [30,31] or not defined as MCI [32], applied individualised non-standardized training [33] or the intervention combined multiple therapies [22,34,35].

### Analysis

A meta-analysis of the RCTs was considered inappropriate as not more than two studies shared the same global outcome measure (Mini Mental State Exam (MMSE) [11,27]), memory specific story or paragraph recall [28,36], and no common mood outcome measure. Outcome measures were similarly heterogeneous in NRCTs and UCTs. The ten trials in MCI were analysed individually with sample characteristics described in Table 1, intervention and outcomes described in Table 2, and effect sizes presented in Tables 3 and 4.

**Table 1:** Sample characteristics of the published randomized controlled trials (RCT) non-randomized controlled trials (NRCT) and uncontrolled trials (UCT) of cognitive training interventions in mild cognitive impairment (MCI)

Citation	Study Design	Type of Training	MCI Sample size (n)	Age (yrs)	Gender (% male)	MMSE (0-30)	Criteria for diagnosis of MCI	Exclusion of co-morbidities
Rapp et al 2002	RCT	Memory strategies	Total n = 19				Petersen (1999)	not reported
			Treatment n = 9	73.3 (6.6)	11%	28 (1.5)		
			Control n = 10	75.1 (7.03)	70%	27.3 (1.8)		

Gunther et al 2003	UCT	Cognitive exercise	n = 19	75-91	21	not reported	Age Associated Memory Impairment, no Dementia, subjective & objective memory complaint,	not reported

Olazaran et al 2004	RCT	Cognitive exercise	Total n = 12		35%*		Flicker (1991)	Physical condition precluding full participation. Illiteracy.
			Treatment n = 8	75.3 (1.05)*		17.2 (3.6)	Neuro-imaging studies	
			Control n = 4	73.3 (1.05)*		21.7 (5.9)		

Belleville et al 2006	NRCT	Memory strategies	Total n = 28				Petersen (2001)	Alzheimer's Disease, alcoholism or "toxicomania", presence of history of psychiatric or neurological disorder, general anaesthesia in the last 6 months
			Treatment n = 20	62.3 (7.3)	not reported	28.9 (1.2)		
			Control = 8	not reported	not reported	not reported		

Cipriani et al 2006	UCT	Cognitive exercise	n = 10	70.6 (6.0)	not reported	28.0 (1.4)	not reported	not reported

Rozzini et al 2007	RCT	Cognitive exercise	Total n = 59	63-7 **	not reported		Petersen (2001)	Geriatric Depression Scale (GDS) > 5
			Treatment CT & ChEI n = 15			26.0 (1.6)	Neuro-imaging studies	Probable or possible Alzheimer Disease, Drug or alcohol abuse or dependence according to DSM-IV criteria, poorly controlled diabetes or other medical condition incompatible with treatment, Previous treatment with ChEIs, Intake of antidepressants during the period of study.
			Control I ChEI n = 22			26.4 (1.9)		
			Control II n = 22			26.8 (1.8)		

Talassi et al 2007	NRCT	Cognitive exercise	Total n = 37				Petersen (1997)	not reported
			Treatment n = 30	76.2 (7.3)	not reported	27.5 (1.4)		
			Control n = 7	76.1 (7.0)	not reported	26.9(1.9)		

Wenisch et al 2007	NRCT	Memory strategies	Total = 24	73.0 (5.5)	33.3	28.1 (.9)	Petersen (2001)	Illiteracy, physical condition preventing full participation, prior cognitive stimulation therapy
			Treatment n = 12					
			Control n = 12 Healthy					

Troyer et al 2008	RCT	Memory strategies	Total n = 50				Petersen (2004)	not reported
			Treatment n = 24	76.0 (5.6)	45%	27.2(1.9)		
			Control n = 26	74.8 (7.7)	45%	28.5(1.0)		

Barnes et al 2009	RCT	Cognitive exercise	Total n = 47				Winbald (2004)	Cerebrovascular disease, starting treatment with ChEIs
			Treatment n = 22	74.1 (8.7)	59.1%	not reported		
			Control n = 25	74.8 (7.2)	60%	not reported		

Values reported as mean (±SD).

*Data given for entire sample of MCI and AD.

** Age range given for entire sample

MMSE: Mini Mental Status Examination

ChEI: Cholinesterase Inhibitor

DSM IV: Diagnostic Manual 4^th ^Edition

**Table 2:** Cognitive training intervention characteristics and cognitive outcomes from randomized (RCT), non-randomized controlled (NRCT) and uncontrolled trials (UCT) in mild cognitive impairment (MCI)

Citation	Cognitive training	Control condition	No. Of sessions/wk	Session length (hrs)	Total no. of sessions/no. wks	Follow-up (mo)	Cognitive outcome measures	Generalisation outcome measures
Rapp et al 2002	Memory strategies (cueing, categorisation, chunking, method of loci) through repetitive exercises and homework drills. Group format.	RCT No treatment	1	2	6/6	6	Domain Specific: Memory Functioning Questionnaire, Memory Controllability Questionnaire, Word list immediate & delayed recall, Story Paragraph immediate & delayed recall, Grocery list immediate & delayed recall, Names and faces immediate & delayed recall	Profile of Mood States

Gunther et al 2003	Cognitive exercise of multi-domain computer-based training with Cogpack (Marker Software, 1992). Format not reported.	UCT None	1	.75	14/14	5	Domain Specific: Six tests from the German Nurnberger-Aging Inventory: TMT, Repeat sentences, Word Lists, Word Pairs, Picture test, Figure test, California Verbal Learning Test- German version (CVLT-G)	None

Olazaran et al 2004	Cognitive exercise with multi-domain pen & paper cognitive exercise drills, within a larger cognitive-motor intervention.	RCT Psycho-social support	2	3.5	100/52	None	Global: Alzheimer's Disease Assessment Scale-Cognitive (ADAS-Cog), Mini Mental Status Exam (MMSE)	Geriatric Depression Scale (GDS), Functional Activities Questionnaire (FAQ)

Belleville et al 2006	Memory strategies of imagery, association, method of loci, and computer-assisted attention training	NRCT Wait list	1	2	8/8	None	Domain Specific: Episodic memory tasks of face naming, word list, story text.	Subjective Memory Questionnaire (QAM), Scale of Well Being

Cipriani et al 2006	Cognitive exercise of multimodal & multi- domain computer-based exercises with NeuroPsychological Training (NPT) (Tonetta, 1995, 1998). Format not reported.	UCT None	4	.75	32/8	None	Global: MMSE Domain Specific: Phonemic and Semantic fluency, Trail Making Test (TMT), Digit Symbol, NPT scores	Rivermead Behavioural Memory Test (RBMT), Geriatric Depression Scale (GDS), Advanced Activity of Daily Living, State and Trait Anxiety (STAI-XI, X2), Short Form Health Survey (SF-12)

Rozzini et al 2007	Cognitive exercise computer-based NPT and Cholinesterase inhibitor (ChEI). Individual format.	RCT Control1 (C1) ChEI Control 2 (C2) No treatment	5	1	72/12	3	Global: MMSE Domain Specific: Story Paragraph, Letter verbal fluency, Semantic verbal fluency, Raven's Coloured Matrices, Rey Complex Figure test (RCFT) copy and recall	GDS-15 items, Neuropsychiatry Inventory (NPI), Basic Activities of Daily Living (BADL)

Talassi et al 2007	Cognitive exercise multimodal & multi- domain computer NPT (Tonetta, 1995, 1998). Group format.	NRCT Physiotherapy	4	.5 to .75	12/3	None	Global: MMSE Domain specific: Digit Span, Rey Complex Figure Test (RCFT) copy and recall, digit Symbol, clock drawing, Phonemic and Semantic verbal fluency	RBMT, Basic and Instrumental Activities of Daily Living (BADL), Neuropsychiatric Inventory (NPI), GDS, STAI, Carer Burden Inventory, physical Performance Test (PPT)

Wenisch et al 2007	Memory strategies categorisation, reality orientation, mental imagery along with executive exercises. Group format.	UCT None	1	1.5	12/12	None	Global: MMSE Domain specific: Logical Memory, Word pair Associate learning, TMT, Semantic and Phonemic verbal fluency.	Goldberg Anxiety & Depression Scale (GADS)

Troyer et al 2008	Memory strategies spaced retrieval, memory book, semantic association, logical location with repeated exercises and homework activities within a larger mixed intervention. Group format.	RCT Wait list	not reported	2	10/25	3	Domain specific: Word List of two-syllable nouns, Digit span, Memory strategy knowledge, Multi-factorial Memory Questionnaire	None

Barnes et al 2009	Cognitive exercise computer-based training of 7 exercises to improve information processing and accuracy of auditory cortex developed by POSIT Science Corporation (San Francisco, CA). Individual home-based format.	3 types of computer activities	5	1.6	not reported	None	Global: Repeatable battery for Assessment of Cognitive Status(RBANS) Domain Specific: California Verbal Learning test, Controlled Oral Word Association Test, Boston Naming Test, TMT, Design Fluency Test, Spatial Span	GDS

**Table 3:** Effect size and study power analysis of memory performance measures, global cognitive function and mood, in trials of cognitive exercises in mild cognitive impairment (MCI)

Citation	Design	Outcome measure	Effect size and 95% confidence Interval	ES > .5 or < -.5	Power of study	> 1 whole number change in test score	Could Type II errors explain lack of significance?
Gunther et al 2003	UCT	Repeat sentence T1	.46 (-.18, 1.11)	No	Single	Yes	Yes
		Repeat sentences T2	.17 (-.50, .83)	No	group	Yes	
		CVLT list immediate T1	.80 (.14, 1.47)	Yes		Yes	
		CVLT list immediate T2	.35 (-3.2, 1.02)	No		Yes	
		CVLT Word lists delay T1	1.21 (-.52, 1.91)	Yes		Yes	
		CVLT Wordlist delay T2	.45 (-.22, 1.12)	No		Yes	

Olazaran et al 2004	RCT	ADAS-Cog	.84 (-.53, 2.21)	Yes	.30	No	Yes
		MMSE	-.10 (-1.43, 1.23)	No	.06	No	
		GDS^	-.33 (-1.23, 1.17)	No	.05	No	

Cipriani et al 2006	UCT	MMSE	.50 (-.39, 1.39)	Yes	Single	No	Yes
		RBMT	.64 (-.26, 1.54)	Yes	group	Yes	
		GDS^	-.33 (-1.21,.55)	No		Yes	

Rozzini et al 2007	RCT	Story recall vs C1	.99 (.29, 1.68)	Yes	.52	Yes	Yes
		Story recall vs C2	.82 (.14, 1.50)	Yes	.77	Yes	
		MMSE vs C1	-.37 (-1.04, .29)	No	.19	No	
		MMSE vs C2	-.58 (-1.25, .09)	Yes	.52	No	
		NPI^ vs C1	-.82 (-1.5, -.14)	Yes	.77	Yes	
		NPI^ vs C2	-.64 (-1.31, -.14)	Yes	.92	Yes	
		GDS^ vs C1	-.26 (-.92, 1.29)	No	.45	No	
		GDS^ vs C2	-.52 (-1.18, 15)	Yes	.54	No	

Talassi et al 2007	NRCT	MMSE	.07(-.76, .89)	No	.14	No	No
		Episodic immediate	.10(-.73, .92)	No	.12	No	
		Episodic delay	.14 (-.96, .68)	No	.09	No	
		GDS^	-.35(-1.18, .47)	No	.20	Yes	

Barnes et al 2009	RCT	RBANS total	0.33 (-.26, -0.92)	No	.29		Yes
		RBANS immediate memory	.38 (-.21,-0.96)	No	.35		
		RBANS delay memory	.53 (-.05, -1.10)	Yes	.55		
		GDS^	No data				

ES: Effect size. Relative ES for RCTs and NRCT, and ES for single intervention group in UCT were calculated by authors of this review as described in the methods analysis

Power analysis calculated by the authors of this review as described in the methods analysis. *Significance of effect size contrast from authors' ES analysis

NRCT: Non-randomised controlled trial

UCT: Uncontrolled trial

T1: Immediate

T2: At follow-up

C1: Control Cholinesterase inhibitor

C2: Control

ADAS-Cog: Alzheimer Disease Assessment Scale-Cognitive

NPI: Neuropsychiatry Inventory

MMSE: Mini Mental Status Examination

**Table 4:** Effect size and study power analysis of memory performance, global cognitive function and mood in trials of memory strategy training in mild cognitive impairment (MCI)

Citation	Design	Outcome measure	Effect size and 95% confidence Interval	ES > .5 or < -.5	Power of study	> 1 whole number change in test score	Could Type II errors explain lack of significance?
Rapp et al 2002	RCT	Word List immediate T1	.33 (-.58, 1.23)	No	.16	Yes	Yes
		Word list immediate T2	-1.18 (-2.24, -.11)	Yes	.71	No	
		Word list delay T1	.88 (-.07, 1.82)	Yes	.57	Yes	
		Word list delay T2	-.36 (-1.36, .64)	No	.16	Yes	
		Paragraph immediateT1	-.03 (-.93, .87)	No	.05	Yes	
		Paragraph immediate T2	-.54 (- 1.54, .47)	Yes	.26	Yes	
		Paragraph delay T1	.38 (-.53, 1.29)	No	.19	Yes	
		Paragraph delay T2	-.14 (-1.13, .85)	No	.08	Yes	
		Profile of mood states	No data				

Belleville et al 2006	NRCT	Word list immediate	.78 (-.06,1.63)	Yes	.75	No	Yes
		Word list delay	.62 (-.22, 1.45)	Yes	.42	Yes	
		Text immediate	.09 (-.73, .91)	No	.07	Yes	
		Text delay	-.42 (-1.25, .41)	No	.25	No	
		Scale of wellbeing	.21 (-.41, .83)	No	.13	Yes	

Wenisch et al 2007	UCT	Story recall immediate	-.71 (-1.54, -.11)	Yes	Single	Yes	Yes
		Story recall delay	.09 (-.71, .89)	No	group	No	

Troyer et al 2008	RCT	Word list T1	.23 (-.82, .35)	No	.18	No	No
		Word list T2	-.02 (-.58, .62)	No	.06	No	

ES: Effect size. Relative ES for RCTs and NRCT, and ES for single intervention group in UCT were calculated by authors of this review as described in the methods analysis

Power analysis calculated by the authors of this review as described in the methods analysis

*Significance of effect size contrast from authors' ES analysis

NRCT: Non-randomised controlled trial

UCT: Uncontrolled trial

T1: Immediate

T2: At follow-up

C1: Control Cholinesterase inhibitor

C2: Control

ADAS-Cog: Alzheimer Disease Assessment Scale-Cognitive

NPI: Neuropsychiatry Inventory

MMSE: Mini Mental Status Examination

CVLT: California Verbal Learning Test

RBMT: Rivermead Behavioural Memory Test

### Study Quality Assessment

There was significant disparity in RCT study quality, with an average CONSORT rating of only 13.5 out of a possible 22 items, with limitations primarily due to poorly explicated methodology and randomization process. The highest scores of 18 and 17 were obtained from a multi-component cognitive exercise intervention [27] and computer delivered cognitive exercise [37] respectively, and the remaining 3 trials were awarded scores of between 9 and 11.

### Cohort Characteristics

Summary descriptions of each study cohort are shown in Table 1.

#### Sample

The ten trials yielded a total of 305 participants, with small cohorts ranging in size from 10 [8] to 59 [11]. All participants were community-dwelling individuals. Recruitment source was variable, with referral from geriatric, psychiatric, memory clinics or neurology units most common [8,10,15,27,37], however frequently no recruitment information was reported [11,12,36]. Disparity in the type and quality of reported demographic information precluded mean calculations. Participants were predominantly women aged in the mid-seventies who had completed secondary school. In some studies, there was an inequality between treatment and control groups at baseline in gender ratios [36], and MMSE scores [27,28]. Limited information was provided regarding health status and medication, and only five studies listed exclusion criteria [8,11,15,27,37].

#### MCI diagnosis

Nine trials applied formal diagnostic criteria to determine the MCI status of subjects. Petersen's MCI criteria were most commonly adopted [8,11,12,15,28,36,38] indicating a predominance of MCI amnesic subtype. No studies operationalized the first criterion of subjective memory complaint [39], although complaints were assessed during interview [28]. In contrast, objective operational measures for the remaining 3 criteria of objective memory impairment, intact general cognitive function, and no functional impairments were uniformly provided. All but two studies, [37,38] provided MMSE scores as a measure of baseline cognitive function, with an average score of 26.32 across eight trials, and range of 28.9 [8] to 17.2 [27] suggesting significant disparity in level of cognitive impairment.

### Cognitive Training Intervention

#### Training format and delivery

Training characteristics are presented in Table 2. Computerized exercises were the most common form of training. The computer programs NeuroPsychological Training (NPT) [40] and Cogpack [41] provided multi-modal and multiple-domain training [10-12,38], whilst the POSIT Science Corporation (San Francisco, CA) program trained only one cognitive domain, i.e. auditory processing [37]. One cognitive exercise trial included pen and paper tasks of repeated 30 minute cancellation, ordering, and mathematical tasks [27].

In contrast memory strategy training involved written and verbal practice of memory strategies including visual imagery [8,15,36], association or categorization [8,15,28,36] and spaced retrieval [28].

Training delivery for both cognitive exercises and memory strategies most often occurred in group [8,12,15,28,36], or combined group and individual sessions [27]. One cognitive exercise trial exclusively involved individual home training [37]. Two memory strategy studies included homework exercises [28,36]. The four memory strategy trials reported that training was supervised by psychologists or neuropsychologists [8,15,28,36].

#### Volume and Duration

Volume of cognitive training measured by hours per week was variable, ranging from 1 hour of memory strategy training [28] to over 8 hours of cognitive exercises [37]. In trials of combined interventions, it was difficult to delineate duration of training from the other intervention components. Duration of exercise training varied from 3 weeks [12] to up to 1 year [27], and memory strategy training ranged from 6 [36] to 26 weeks [28]. Overall mean volume of training (sessions/week × number of weeks) was 8 sessions for memory strategies and 57.5 sessions for cognitive exercises.

#### Combined intervention

Interpretation of results was confounded in all four memory strategy trials [8,28,36], as the predominant memory strategy training was augmented with other interventions that were not controlled for in the comparison group. For example, memory strategy training was combined with occupational therapy and behavioural training [36], life-style education [28], computer assisted attention training [8], and executive exercises [15]. Only one cognitive exercise trial combined other therapy, motor and daily living function training [27].

#### Outcome Measures

There was considerable variability in the type and quality of outcome measures used in each study, limiting the extent to which the efficacy of cognitive training in MCI could be evaluated. The types of outcome measures employed can be broadly classified into training-specific measures, domain-specific cognitive measures (memory, attention, executive function, and speed), global cognitive measures, and secondary generalization measures of function and emotional and behavioural status. Word lists and story or paragraph recall were the most common domain-specific outcome measures. There were no measures of quality of life, and incident dementia was not reported in any trial.

### Effect Size Analysis

Eight of ten studies reported improvement in at least one cognitive outcome (see Tables 3 and 4). Specifically, relative effect sizes (ES) varied between moderate (ES 0.3 - 0.5) to large (> 0.5) on measures of objective memory performance in four of five RCTs (3 cognitive exercise trials [11,27,37] and 1 memory strategy trial [36]), one of three NRCTs [8], and both uncontrolled cognitive exercise trials [10,38]. However, many results were not statistically significant and so post-hoc power calculations were used to assess the rate of probable Type II errors. All RCTs and NRCTs were found to be underpowered (power less than 80%) for the reported memory outcomes.

Effect sizes on memory outcomes were typically greater for the randomized cognitive exercise trials with relative effect sizes on memory outcomes ranging from .10 to 1.21. In contrast relative effect sizes on memory outcomes following memory strategy training ranged from .88 to -1.18. On tests of text recall, this difference appeared strongest. For example, Rozzini et al 2007 found relative ESs that ranged from ES = .82 to ES = .99 based on story recall [11], whilst a study of memory strategy training based on paragraph recall failed to find positive training effects (ES = -.03 to ES = -.54 [36]).

Two RCTs of memory strategy training [28,36] have yielded mixed ES on memory. These studies found no evidence of generalization, with effects being restricted to training-specific outcome measures. Although persistence of strategy use was reported [28], memory performance deteriorated over time [28,36]. In addition, trained subjects had less improvement at six months than control subjects (ES = -1.18) [36]. Overall, large effects (ES > .05) were found for 50% of the memory outcomes in cognitive exercise trials compared to 37% of memory strategy outcomes.

Improvements in mood following cognitive exercises were also found. Cognitive exercises led to a reduction in depressive symptoms in both RCT trials [11,27], suggesting that training benefits may include improved mood. Furthermore a large, significant and clinically relevant reduction in psychiatric and depressive symptoms found after cognitive exercises compared to Cholinesterase inhibitors (ChEI) treatment (ES = -.82) [11], suggesting that cognitive exercise training may have adjunctive benefit to this medication. The two randomized memory strategy trials did not include outcome data on measures of mood [28,36].

Results from the NRCT and UCTs indicated larger effects and clinical benefits following computer-based exercises compared to pen-and-paper memory strategy training across a number of domain specific, global cognitive, and mood function measures [10,12,38]. For example, an UCT [10] and NRCT [12] cognitive exercise trials both resulted in a small reduction of depressive symptoms. Belleville's 2008 NRCT of memory strategy training yielded a large positive result on word list recall however, interpretation is difficult due to lack of randomization and the use of multiple interventions, including computer exercises, with the relative contribution of each intervention not discernable.

Greater volume of training was associated with greater effect on memory outcomes following cognitive exercises (60 sessions ES = .82 and .99 [11], compared to 12 sessions ES = .10 [12]), but not for memory strategy training (6 sessions ES = .88 [36], compared to 10 sessions ES = .23 [28]). Combined memory strategy with attention training [8] was associated with large and significant benefit (ES = .78) following only 8 sessions. Supervision of training was reported in the four memory strategy training trials [8,15,28,36], making it impossible to evaluate the benefit of supervision compared to no supervision in strategy training, and was not reported upon in the computer-based cognitive exercises trials. Training was provided in group format with the exception of two trials [11,37] therefore comparison of benefit between individual versus group format was not feasible.

#### Longitudinal Follow-up

Three RCT studies examined persistence of effect with longitudinal follow-up. Unexpectedly, memory strategy training was associated with decreases in objective memory performance at three [28] and six months [36] post training compared to control. By contrast, improvements in function were evident following cognitive exercises at three months [11]. However, an uncontrolled cognitive exercise trial found the benefit of training did not persist at five months follow-up [38].

## Discussion

This systematic review applied defined criteria to identify original cognitive training studies that investigated cognitive efficacy in preclinical MCI subjects. In addition to RCTs, less robust NRCT and UCT designs were included in this review to exhaustively review the existing literature, the relationship of study quality to outcomes achieved, and identify gaps in the literature. Nonetheless, our systematic review identified only ten trials of cognitive training using either cognitive exercise or memory strategy approaches, of which half were RCTs of low to moderate quality, with significant heterogeneity. Despite these limitations, certain patterns did emerge. Moderate-sized effects were found on memory performance and global cognitive measures in a majority of studies, with computer-based cognitive exercise studies exhibiting an increased frequency of stronger effect sizes, and enhanced generalization of benefits, compared to memory strategy training. However, most studies were underpowered for the effects achieved, and so individual results were often insignificant. Furthermore, three trials [27,28,36] included additional intervention components so that the unique benefit of cognitive training is difficult to assess. Overall, the field is nascent and further high quality RCTs are of critical importance.

### Defining and Classifying Cognitive Training

The literature regarding cognitive training has so far suffered from a variable definition of intervention, as well as the frequent use of multiple interventions without appropriate controls, thereby accounting for the inconsistent results. For example, two recent meta-analyses of cognitive training in healthy older adults [23,42] drew different conclusions, mainly because inclusion criteria varied, with only three studies being common to both analyses. In MCI, previous reviews have included mixed interventions without clearly delineating between cognitive exercise and memory strategy training, and also included different trials [4,43,44]. Mixed interventions only add to the confusion. For example, a recent randomized control trial nominally compared 'cognitive training', comprising health education, meditation, memory strategy training and problem solving strategies, with 'cognitive stimulation', itself comprised of reality orientation, quiz games and problem solving activities [45]. In the area of cognitive training in established dementia, reviews have similarly produced mixed results: two reports found little support for efficacy [46,47], but a subsequent review which distinguished between restorative cognitive training and compensatory strategies found that cognitive training appeared to be of modest benefit [48]. If general stimulation activities and rehabilitative compensation are excluded, perhaps, cognitive training can be examined appropriately as a unique stand-alone intervention. In addition, we here distinguished between cognitive exercises and memory strategy training because of fundamental differences in approach and intent.

### Evidence from trials of Cognitive training in MCI

Cognitive exercise involving multiple cognitive domains appears to demonstrate greater efficacy than uni-modal memory strategy training. Multi-domain exercises provide a broader range of cognitive challenges to directly stimulate plasticity, and in several studies has resulted in improved global cognitive function [11,12,27,49]. By contrast, little evidence was found for the efficacy of memory strategy training in MCI which was consistent with outcomes from a recent meta-analysis in healthy and MCI subjects that found training effects were equivalent to those seen in active controls [50]. Memory strategy training may have limited generalizability to overall cognitive function, perhaps because it has a very specific nature and reliance on subjects' ability to appropriately apply acquired strategies. Since complex mental activity induces a number of central nervous system adaptations, including neuro-protective, plastic, trophic and compensatory mechanisms [50], multi-domain cognitive exercise may be better suited to stimulating these neuroplastic brain changes. Neuroimaging studies, for example, are beginning to isolate functional, structural and biochemical changes that accompany cognitive training [Suo & Valenzuela, in press]. Further research is required that directly compares memory strategy training to multi-modal cognitive exercises, as well as single- versus multi-domain cognitive exercises. For the moment it is important to recognise that firm conclusions cannot be made because of the prevalence of mixed interventions and overall limited quality studies.

High volume cognitive exercise appeared to result in greater benefit than lower volumes of training, although no dose-response studies were identified. Very frequent training for twelve weeks led to greater effect on memory [11] than longer, less regular training [27,38], and cognitive exercise studies generally had a higher frequency of training sessions at four [10,12] or five sessions per week [11,37]. The observation of greater efficacy from cognitive exercise than memory strategy training may therefore simply represent a function of training volume, and so volume-matched comparative studies are required. Meta-analysis of cognitive training in healthy adults has suggested that 2-3 month training periods may have persistent protective benefit [23] however current findings in MCI suggest that frequency and total volume of sessions are also important. Accordingly, it is possible that training needs to reach a 'critical threshold' in order to produce sufficient adaptive neurobiological changes. However, given the variability observed, it is not yet possible to determine the minimum required frequency, volume or duration of cognitive training and explicit dose-response trials in MCI that are needed.

### Clinical Role of CT in Primary and Secondary Prevention

Three stages of AD prevention have been identified: *primary prevention *to reduce disease incidence in cognitively normal individuals; *secondary prevention *to slow progression of pre-clinical disease to clinical disease (often translating to reduction of MCI 'conversion' to dementia); and *tertiary prevention*, the reduction of disability due to cognitive symptoms in diagnosed patients [51]. Cognitive training can be applied to each of these stages, and it is proposed that the type of training intervention should vary depending upon the prevention stage. Cognitive exercise is designed to improve function through neuroplastic mechanisms and has been shown to produce positive effects in healthy adults [23] thereby consistent with a primary prevention goal. This current review suggests cognitive exercise also has promise for enhancing cognitive function in MCI [10-12,37,38], and may slow decline in at risk individuals, consistent with secondary prevention. As the syndrome of MCI may have different aetiologies [25], it is likely that individuals with different subtypes of MCI may respond differently to treatment. For some, training in strategies to compensate for memory difficulties may have additional value, and consequently combined cognitive exercise and memory strategy training may be optimal. However, comparative trials are required and there remains a lack of longitudinal research. The effectiveness of cognitive exercise as a tertiary prevention in those with established AD is likely to be modest [48], although a recent trial of computer-based exercises found delayed progression of disease by the end of training compared to controls [52]. For tertiary prevention, compensatory rehabilitative memory strategy training approaches that target disability maybe appropriate.

Computer-delivered interventions are rapidly becoming popular. Computerized cognitive exercise has been successfully implemented across the age spectrum and research suggests that older adults are often the fastest growing users of computer and internet technology [53]. Computer delivered exercises may provide primary and secondary prevention and be accessible to a wide number of individuals. On the other hand, it may be more appropriate to deliver tertiary compensation strategies by traditional pen and paper methods that minimize memory load. Finally, across all of these types of interventions and prevention stages, quantifying the 'real world' significance of cognitive training interventions on instrumental activities of daily living and quality of life will be vital and so far has not been addressed.

## Conclusions

Further research is urgently required in order to substantiate the efficacy of cognitive training as a therapeutic intervention in MCI. It is vital to clearly distinguish between various cognitive interventions and differentiate between training exercises and memory strategies. This review suggests cognitive exercise may be effective at enhancing cognitive outcomes, but several limitations have been identified which precludes firm conclusions. All trials have been small and generally underpowered, and thus larger and more diverse cohorts are needed. Notably, only two RCTs [11,37] to date have examined the isolated benefit of cognitive exercise, whilst the other three included co-interventions. Importantly, no significant negative or adverse effects of cognitive training have been found, in marked contrast to drug trials in MCI, where side effects and high dropout rates are commonly reported [47].

The following recommendations are intended as an indicative rather than exhaustive list, and demonstrate the challenges and opportunities for the field:

1. Employ a rigorous cognitive training definition, and distinguish between cognitive exercises and memory strategy training in abstracts and reports. A consistent operational definition will facilitate the appropriate comparison of effects between interventions, and provide more precise information for program development and research.

2. RCTs with active control conditions (sham training) are required to control for non-specific effects. Similarly, in multi-modal and combined interventions, additional study arms are needed.

3. Assess generalization by testing cognitive, behavioural, quality of life, functional, mood, and psychological wellbeing outcomes.

4. Compare the effects of training volume and duration, as well as investigate dose-response relationships.

5. Proximal and longitudinal follow-up assessments are needed to determine the persistence of effects and begin characterising the temporal course of putative benefits.

6. Comprehensive description of inclusion criteria and sample descriptors are needed in order to control for the potential heterogeneity in MCI aetiology.

## Competing interests

The authors declare that they have no competing interests.

## Authors' contributions

NG and MV conceived of the study and participated in its design and co-ordination. NG conducted the search, NG and MV completed data extraction; and NG and MV conducted data analysis. NG drafted the manuscript and MV, PS and MFS revised and edited the manuscript. All authors read and approved the final manuscript.

## Pre-publication history

The pre-publication history for this paper can be accessed here:

http://www.biomedcentral.com/1471-2318/11/55/prepub
